# Microfluidic Cultures of Basal Forebrain Cholinergic Neurons for Assessing Retrograde Cell Death by Live Imaging

**DOI:** 10.21769/BioProtoc.5149

**Published:** 2025-01-05

**Authors:** Srestha Dasgupta, Mansi A. Pandya, Wilma J. Friedman

**Affiliations:** Department of Biological Sciences, Rutgers University, Newark, NJ, USA;

**Keywords:** Microfluidics, Basal forebrain, Live imaging, Cell death

## Abstract

Neurons are highly polarized cells, with axons that may innervate distant target regions. In the brain, basal forebrain cholinergic neurons (BFCNs) possess extensive axons that project to several target regions such as the cortex, hippocampus, and amygdala, and may be exposed to a specific microenvironment in their axon targets that may have retrograde effects on neuronal health. Interestingly, BFCNs express the pan-neurotrophin receptor p75NTR throughout life while also concomitantly co-expressing all Trk receptors, making them capable of responding to both mature and precursor neurotrophins to promote survival or apoptosis, respectively. Levels of these trophic factors may be modulated in the BFCN axon or soma microenvironment under neurodegenerative conditions such as seizure and brain injury. In this protocol, BFCNs are established in microfluidic devices for compartmental culture, with the aim of studying the effects of axon- or soma-specific stimulation of BFCNs for an in vitro representation of distal axon vs. soma environments as seen in vivo. This study further establishes a novel method of tracing and imaging live BFCNs exposed to stimuli in their distal axons with the aim of assessing retrograde cell death. The in vitro compartmental culture system of BFCNs that allows live imaging may be applied to investigate various effects of axon- or soma-specific stimuli that affect BFCN health, maintenance, and death, to model events that occur in the context of brain injury and neurodegenerative disorders.

Key features

• Separation of axons and soma of basal forebrain primary neurons in vitro using microfluidic chambers.

• Compartmental/localized treatment of axons or somas of BFCNs.

• Live imaging of retrogradely labeled BFCNs to assess cell death.

## Background

Neurons may possess long axons that project to distal areas and respond retrogradely to stimuli limited to their axonal microenvironment. Basal forebrain cholinergic neurons (BFCNs) send extensive and long axonal projections to several distal brain areas, such as the cortex, to regulate cognitive functions such as attention, emotion, and memory [1]. Interestingly, throughout their life, BFCNs express the pan-neurotrophin receptor p75NTR as well as all three members of the Trk family of receptor tyrosine kinases. Neurotrophins are essential for BFCNs’ survival, maintenance, differentiation, and function [2]. Neurotrophins in their precursor and mature forms activate different signaling cascades, which lead to contrasting cellular responses. Proneurotrophins are cleaved to form mature neurotrophins, which promote a pro-survival response through Trk signaling [3–6]. In contrast, under conditions of brain insult such as seizures or traumatic brain injuries (TBI), proneurotrophins (proNTs) bind p75NTR and sortilin to promote apoptosis in BFCNs [7]. Interestingly, as BFCNs express all the neurotrophin receptors, these neurons are capable of responding to a changing balance of pro vs. mature neurotrophins. To add to the complexity, the dynamic shift in pro vs. mature neurotrophin balance may occur at the BFCN neuronal target regions such as after cortical injury [8]. Although it has been established that p75NTR promotes BFCN degeneration in mass cultures and after seizure conditions in vivo [7], given the loss of BFCNs after cortical injury it is important to answer whether p75NTR can promote BFCN degeneration retrogradely as well, especially when the BFCN soma is unexposed to induced proneurotrophins [8].

 This protocol has been applied by Dasgupta et al. [8] to establish whether the changes in the neurotrophic environment of BFCN axon terminals after TBI have a retrograde effect on survival of these afferent projections by live imaging and cell death analysis of in vitro compartmentally cultured BFCNs. Silicone microfluidic devices allow for the segregation of neuronal axons and soma as well as for compartmental manipulation with localized treatments [9], having been used extensively on several neuronal cell types to investigate a myriad of cell biology questions [10]. Moreover, the transparent material allows for labeling and imaging of live tracers that indicate dying cells after treatments. Major studies have established retrograde degeneration through p75NTR in peripheral neurons [11,12], but this protocol helped establish the role of p75NTR and proneurotrophins on BFCN retrograde degeneration, adding to the evidence that suggests that brain injury induced elevation in cortical proneurotrophin leads to p75NTR signaling in BFCNs initiated at the axon terminals to promote retrograde degeneration [8]. Overall, this protocol can be applied to study cell death and other cellular responses to compartmental stimulation of BFCNs by live imaging and can also be adapted to other neuronal populations.

## Materials and reagents

**Biological materials**

1. C57BL/6, strain# 000664 mice were purchased from The Jackson Laboratory and mated in-house. Timed pregnant mice at a pregnancy age of E15 were used for the basal forebrain dissection.

**Reagents**

1. Poly-d-lysine (Sigma-Aldrich, catalog number: A-003-M)

2. Glucose (Sigma-Aldrich, catalog number: G7021)

3. Transferrin (Sigma-Aldrich, catalog number: T4382)

4. Insulin (Sigma-Aldrich, catalog number: I5500)

5. Putrescine (Sigma-Aldrich, catalog number: P7505)

6. Selenium (Sigma-Aldrich, catalog number: S-5261)

7. Progesterone (Sigma-Aldrich, catalog number: P0130)

8. Penicillin and Streptomycin (10,000 IU) (Sigma-Aldrich, catalog number: P4333)

9. Minimum essential medium (MEM) (Gibco, catalog number: 11095080)

10. Ham’s F-12 media (Gibco, catalog number: 11765054)

11. B-27^TM^ Plus supplement (50×) (Gibco, catalog number: A3582801)

12. Sterile PBS (1×) (Gibco, catalog number: 10010023)

13. Cholera toxin subunit B (recombinant), Alexa Fluor^TM^ 488 conjugate (CTB) (Invitrogen, catalog number: C34775)

14. Propidium iodide (PI) (Molecular Probes, catalog number: P1304MP)

15. Sylgard 184 Silicone Elastomer kit (Dow, catalog number: 4019862)

16. Isopropanol (Sigma-Aldrich, catalog number: I9030)

17. Paraformaldehyde (Sigma-Aldrich, catalog number: P6148)

**Solutions**

1. Serum-free media (SFM) (see Recipes)

**Recipes**

**1. Serum-free media (SFM)**

1:1 mixture of Eagle's MEM and Ham's F-12 supplemented with glucose (6 mg/mL), progesterone (20 nm), putrescine (60 μm), transferrin (100 μg/mL), selenium (30 nm), penicillin (0.5 U/mL), and streptomycin (0.5 μg/mL) [4]. Alternatively, you may use commercially available Neurobasal media.

**Laboratory supplies**

1. Sterile Petri plates (60 mm) (Nunc^TM^ EasYDish^TM^ Dishes, catalog number: 150462)

2. Sterile surgical tweezer set (DUMONT Fine Science Tools, catalog number: 11251-10)

3. Surgical scissors (Millipore Sigma, catalog number: S3146)

4. Surgical scalpels (WPI Scalpel Handle, model: #4, catalog number: 500237-G)

5. Sterile scalpel blades (Cincinnati Surgical, catalog number: 00SME11)

6. Spatula (Sigma-Aldrich, catalog number: Z513342)

7. Rapid core puncher, ID 6.0 mm, OD 6.5 mm, white (Well Tech, Ted Pella Inc, catalog number: 15115-12)

8. Hemocytometer (Fisher Scientific, catalog number: 02-671-51B)

9. Sterile serological pipettes (VWR, catalog number: 89130-898, 10 mL)

10. Sterile glass coverslips (25 mm) (Deckglaser, Carolina coverglass, catalog number: 633037)

11. Falcon tube (15 mL) (Fisher Scientific, catalog number: 14-959-53A)

## Equipment

1. Laminar flow culture hood (Edgegard) (GMI, catalog number: 8038-30-1041)

2. Nikon SMZ1000 dissecting microscope (Nikon, catalog number: SMZ1000-P)

3. CO_2 _incubator (Sanyo, model: MCO-17AC)

4. Isotemp oven (Fisher Scientific, catalog number: 13-247-750G)

5. Vertical rotating mixer (Atlantis Bioscience, catalog number: VM-80)

6. Scienceware^®^ vacuum desiccator (Sigma-Aldrich, catalog number: Z119016)

7. Confocal microscope for live imaging (Zeiss, model: LSM 510 microscope)

## Software and datasets

1. Fiji: ImageJ (Open Source; https://github.com/imagej) (https://imagej.net/software/fiji/)

## Procedure

**A. Basal forebrain primary neuron compartmental culture**

1. Microfluidic device casting

a. Preparation of positive relief microfluidic mold

i. To create microfluidic devices in-house, Polydimethylsiloxane (PDMS), a silicone elastomer (Sylgard 184), is cast on a positive relief resin microfluidics mold with multiple chambers etched (adapted from Harris et al. [13]; Taylor et al. [9]).

ii. Molds can be reused to cast devices, and therefore need deep cleaning before every casting process. To clean the mold, release pressurized air on the positive relief surface in 5–10 repeats vigorously to remove the remaining PDMS from earlier usage. A thin tube is attached to the pressurized air inlet to which a 1,000 mL pipette tip is attached, and the pipette tip is moved over the surface to create sharp blasts of air; the purpose is to remove any debris from the mold.

iii. Measure the mold volume by adding DI water into the mold area to fill it completely; then, remove the water into a 50 mL Falcon tube. Retain this information for the PDMS preparation and casting step.

iv. Fill the mold area with isopropanol. Put the mold on a shaker at medium speed for 20 min for the isopropanol wash.

v. Remove isopropanol and dry the mold uncovered in an oven at 70 °C for 10 min or until completely dry.

vi. Cover the mold to avoid any particulates adhering to the mold.

b. PDMS preparation and casting

i. Sylgard 184 comprises a PDMS base and a PDMS curing agent, which, when combined, produce the silicone elastomer.

ii. Combine PDMS base and PDMS curing agent in a 50 mL Falcon tube in a 10:1 ratio. Use the precalculated mold volume as a reference to the total volume of the PDMS mixture to be prepared.

iii. Place the mixture on a rotator at low speed for 20 min or until the mixture becomes homogenous.

iv. Pour the PDMS mixture into the clean molds, start at a corner, and let the mixture flow across the surface to fill the entire mold.

v. To remove air from the mixture, place the mold with the mixture in a vacuum desiccator at full suction power for 4–5 h.

vi. Assess the progress of the desiccation by observing the number of bubbles visible in the mixture in the mold every 2 h.

vii. Once the PDMS appears clear and has no bubbles left, gently disassemble the desiccator to ensure the PDMS mixture does not spill out of the mold, as it will still be in a liquid state.

viii. Remove the mold with PDMS and transfer it to an oven at 70 °C for baking. Bake the PDMS for 48 h and keep the mold uncovered.

c. Cleaning and preparation of casted PDMS

i. After 48 h of baking, remove the mold from the oven, cover the mold, and let it cool to room temperature.

ii. Use a spatula with a flat tapered end and a sharp scalpel to extract the solid PDMS from the mold. Start by releasing the edges by cutting with the scalpel, then release the PDMS surface facing the positive relief of the mold with the spatula, and gently peel off the entire PDMS.

iii. Based on the mold structure, each PDMS casting produces nine microfluidic chambers. At this stage, all the chambers are present on the PDMS as one block and need to be separated into individual devices.

iv. Use reusable biopsy punchers (size No. 6) as shown by dotted lines in Figure 1a–c to punch out four holes on the edges of the microfluidic channels of each chamber connected by the microgroove, two on either side, just covering the edges so that an open flow of media will be maintained in both channels.

v. Carefully, cut out individual chambers using the scalpel. Cut the corners of the devices so that the chambers fit 25 mm coverslips optimally and have no overhangs.

vi. Clean out loose PDMS in the chambers using scotch tape on the negatively etched PDMS surface.

vii. Further clean the chambers by washing with 70% ethanol for 10 min.

viii. Dry the chambers at 70 °C for 10 min and store in a sterile Petri dish.

2. Microfluidic chamber preparation for cell culture

a. Microfluidic devices created in-house have two channels connected by microgrooves of 450 μm length, with two compartments on either end of the channels that work as media reservoirs.

b. Perform all further steps in the laminar flow hood to maintain sterility.

c. Prepare individual microfluidic devices a day before cell culture by sterilization with 70% ethanol.

d. Prepare SFM a day before culture (see Recipes).

e. Place individual glass coverslips in 35 mm Petri dishes. Precoat glass coverslips for culture a day in advance with 1 mL of poly-d-lysine (0.2 mg/mL) and maintain overnight at a 37 °C, 5% CO_2_ cell culture incubator.

f. On the day of the culture, remove poly-d-lysine from coverslips by suctioning carefully, aiming to remove all the solution but not scratch the coated surface.

g. Ensure sterilized microfluidic devices are completely dry as well. Place the negatively etched surface of the microfluidic device on the coverslip with forceps, while pressing gently at each corner to ensure a water-tight seal.

**Figure 1.**
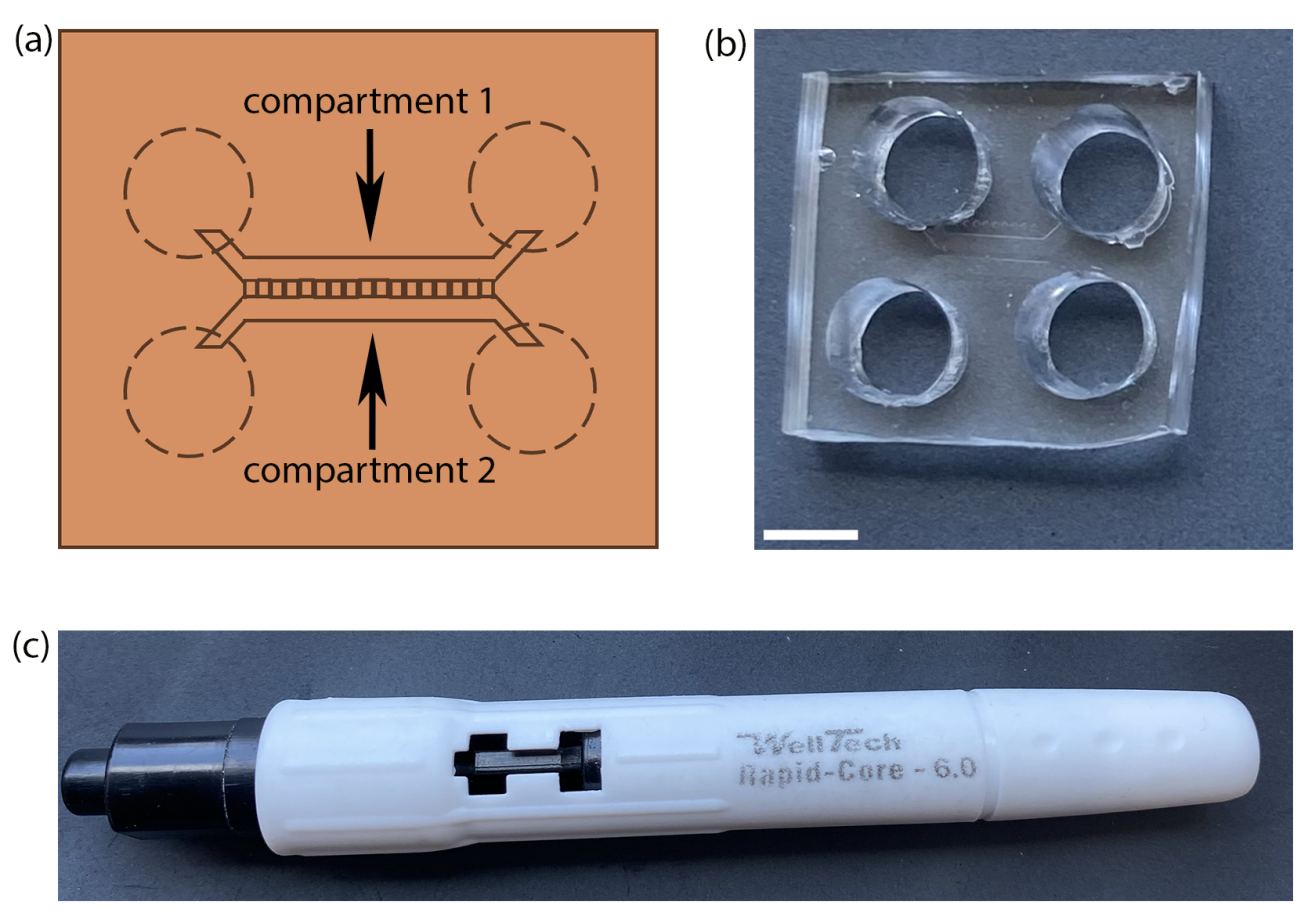
Preparation of microfluidic device by PDMS casting. (a) Schematic of a microfluidic chamber with microgrooves connecting the two compartments. Dotted lines represent the positions to punch holes into the chamber. (b) A microfluidic device made in-house by PDMS casting and preparation using (c) a well puncher of 6 mm inner diameter. Scale bar = 5 mm.

**Figure 2.**
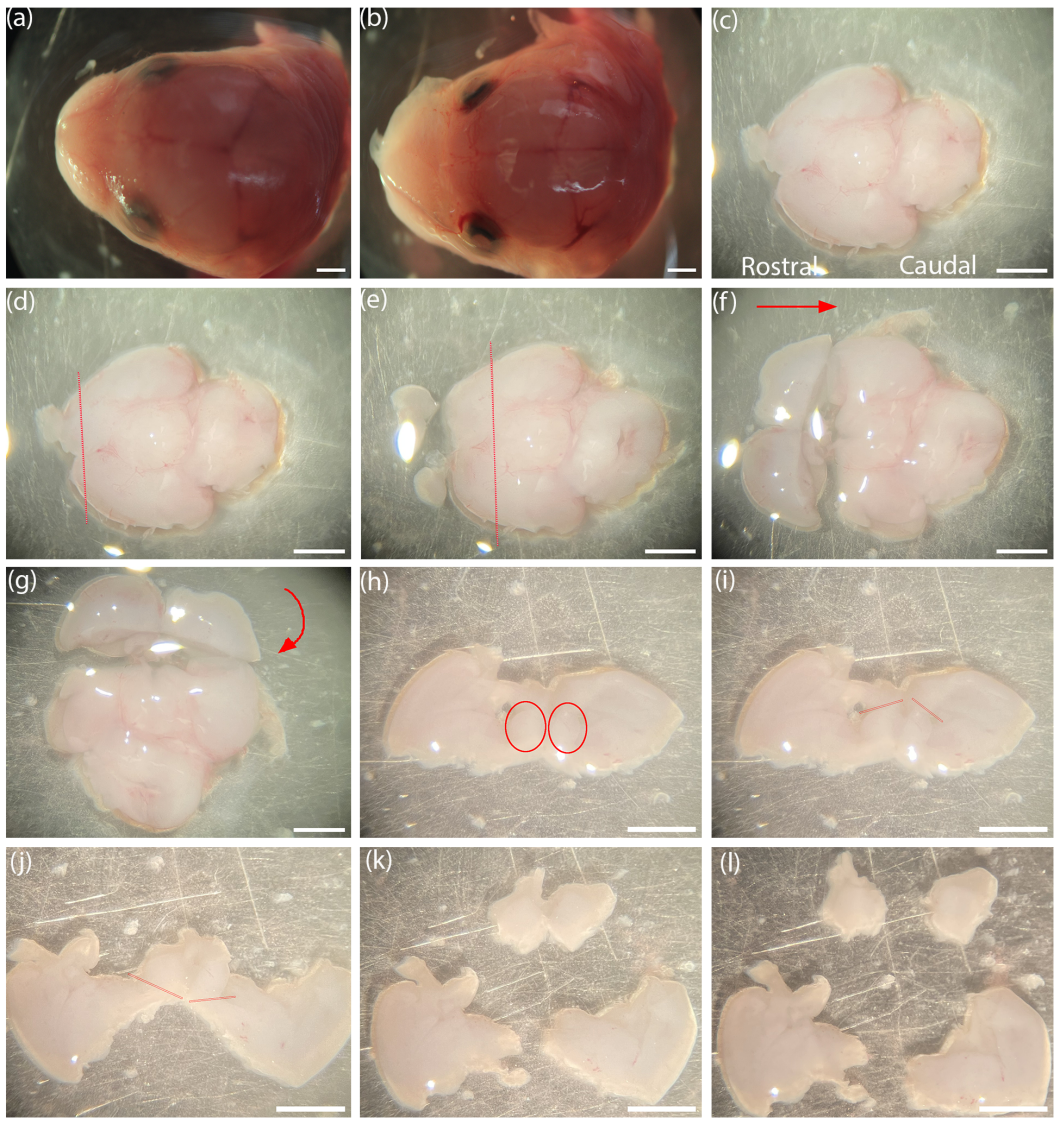
Step-by-step basal forebrain dissection captured using the Nikon SMZ1000 dissecting microscope. (a, b) An E15 mouse brain with and without the skull. (c) Mouse brain with the ventral side up, with rostral to caudal ends facing left to right. (d) Demarcations (red) for coronal incision to remove olfactory bulbs and anterior poles. (e) Demarcations for coronal incision at rostral edge of hypothalamus. (f) Red arrow indicates turning the plate 90° clockwise so that the rostral aspect faces anterior. (g) Red curved arrow indicates laying down the coronal slab at 90° toward the posterior direction such that the rostral aspect will face upward. (h) Coronal slab showing the basal forebrain (red circles). (i) Demarcations (red) for dorsal incision at lateral ventricles of both hemispheres. (j) Demarcations (red) for ventral incision to separate the basal forebrain and medial septum. (k, l) Separated basal forebrains of both hemispheres. Scale bar = 1 mm.

3. Basal forebrain culture

a. Collection of E15 mouse embryo

i. Euthanize a pregnant mouse while the fetuses are at embryonic day 15 (E15) by exposure to CO_2_.

ii. Sterilize by soaking in 70% ethanol for 5 min.

iii. To remove fetuses under sterile conditions by C-section, make a midline incision in the abdomen to sever the skin using a thick scissor from the surgical tool kit. Sever the muscle along the same incision to expose the embryos.

iv. Remove the embryos using a small scissor and forceps and place in a 20 cm sterile Petri dish with PBS. Maintain the Petri dish on ice.

b. Basal forebrain dissection

i. To collect the basal forebrains, place embryos in SFM to dissect as described below (adapted from Friedman et al. [4]) in the laminar flow hood.

ii. Remove each embryo from its amniotic sac with two straight-edged fine forceps. Separate the heads with the forceps, place in another Petri dish with PBS, and place on ice.

iii. Place a Petri dish with PBS under the dissecting microscope. Place a separated head upright using another set of fine forceps (Figure 2a). Anchor the head on the ventral side with one forceps while clasping onto the top of the skull (gelatinous and soft at this age) with the other forceps. Let go of the ventral anchor and clasp an adjacent section of the skull. Gently peel off the skull with the two forceps with a pull in opposite directions.

iv. Successfully removing the skull will reveal the brain (the shiny translucent layer covering the brain will have been removed) (Figure 2b). Gently turn the head to its side and slide out the brain. Cut the connections at the olfactory bulbs to free the brain from the head with a pair of sharp scalpels.

v. Align the brain with the ventral aspect facing upward and the olfactory bulbs on the rostral aspect of the brain to the left (Figure 2c).

vi. Cut coronally to remove the frontal poles of the cortex along with the olfactory bulbs (Figure 2d).

vii. Cut at the demarcation of the rostral edge of the hypothalamus to generate a thick coronal section (Figure 2e).

viii. Turn the thick coronal section at 90°, laying the section down such that the rostral cut side is facing upward (Figure 2f, g).

ix. In the coronal section, make incisions at the dorsal tips of the lateral ventricle on both hemispheres (Figure 2h, i) and at the ventral surface of the brain to separate the medial septum, together with portions of the diagonal band of the basal forebrain (Figure 2j). Collect the separated septum and diagonal band of the basal forebrain (Figure 2k, l) and clear the rest of the tissue debris to one side of the Petri dish.

x. Repeat steps v–ix to collect the basal forebrain from all the embryos into a separate small Petri dish (30 mm) with SFM.

xi. Cut the collected tissue into small pieces with the scalpels in a low volume of media in a separate Petri dish (500 µL to 1 mL), aiming to have a high concentration of cells (approximately 2–3 million cells/mL). Dissociate cells using a Pasteur pipette by trituration (repeated pipetting) approximately 20–30 times until cells are dissociated into a cloudy suspension.

c. Plating BFCNs in microfluidic devices

i. Count cells using a hemocytometer and pipette 50,000 cells in a pressure pulse through one side of the microfluidic chamber that should populate the channel. This channel will be considered the soma compartment. The average cell yield per basal forebrain (both hemispheres combined) per embryo is about 800,000 cells.

ii. Basal forebrains pooled from several embryos dissociated in 500 μL–1 mL of media should yield an optimal concentration of cells (2–3 million/mL) to plate 50,000 cells in a concentrated media volume (~10 μL).

iii. Prepare SFM + 1% B27 to plate and maintain cells.

iv. After 5 min of plating cells, add 200 μL of SFM + 1% B27 media to each reservoir of the soma compartment. Add 100 μL of SFM + 1% B27 in the reservoirs of the opposite side, which is considered the axon compartment.

v. Maintain a volume gradient between the soma and axon side to promote neurite growth through the microgrooves to the axon compartment for five days at 37 °C and CO_2_ (5%) to obtain compartmentalized BFCN cultures.

vi. Add 50 μL of SFM + 1% B27 to all compartments every alternate day after culture to account for media evaporation from the microfluidic devices.

**B. In vitro labeling and live imaging of BFCNs in microfluidics**

1. Retrograde labeling of live neurons

a. Prepare a working solution of a retrograde tracer, Alexa 488 labeled choleratoxin B (CTB, 1 μg/mL) in SFM.

b. At 5 days in vitro (DIV), treat the axon compartment with the SFM + CTB working solution and incubate for 20 min in the incubator.

c. After 20 min, wash the axon compartment twice with SFM + 1% B27 media to remove any excess tracer, then add 100 μL of media to maintain axons.

d. Incubate for at least 5 h to allow CTB to be transported retrogradely to the somas of BFCNs that have extended their axons into the distal compartment.

e. Prior to compartmental stimulation of the BFCNs with desired treatments, replace the soma media with a working solution of SFM + 1% B27 + PI (1 μg/mL) for 10 min. PI will label the nuclei of dying neurons with an excitation maximum of 535 nm. PI does not need to be washed out and remains in the media throughout the experiment.

f. Maintain the media volume gradient between the soma and axon compartment for the duration of the treatment as well.

**Figure 3.**
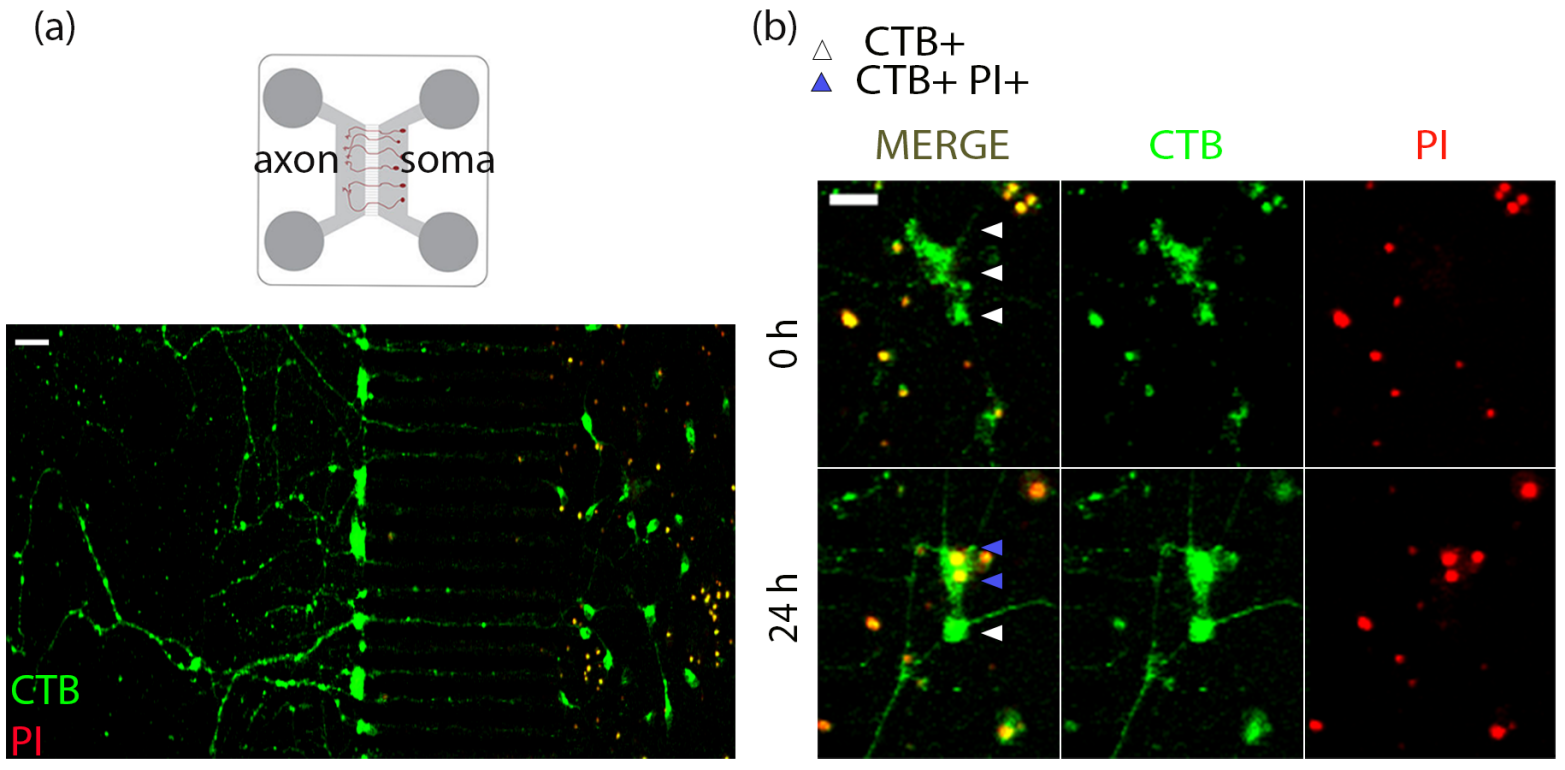
Live tracing and imaging of basal forebrain cholinergic neurons (BFCNs) in microfluidic cultures to study retrograde cell death. (a) Representative images of E15 mouse basal forebrain neurons cultured in microfluidic chambers for 5 DIV labeled with a retrograde tracer CTB Alexa 488. Before live imaging, propidium iodide (PI) was added to the soma compartment to identify dying neurons. Scale bar = 50 μm. (d) After 24 h of treatment, CTB+ (white arrowheads) cells are counted as surviving cells, whereas CTB+PI+ (blue arrowheads) cells are counted as dying cells. Scale bar = 20 μm.

2. Live imaging of microfluidics to assess cell death

a. For live imaging, cells need to be maintained at a constant temperature (37 °C) and CO_2_ (5%) for the duration of imaging for the experiment.

b. Set up the microscope equipped for live imaging with these settings before adding treatments to the cell culture.

c. Set up the microscope at 10× objective. Use the brightfield channel to locate the microgrooves such that both the axon and soma compartments are in the optical field (Figure 3a).

d. CTB 488 has an Alexa 488 fluorophore attached to it, and PI fluoresces at a 555 nm wavelength once it enters the nuclei of dying cells. Set up the 488 and 555 channels at a low laser power (5%–10%) to avoid phototoxicity optimized based on signal from the sample. Adjust digital offset and gain if necessary, using the range indicator. Set camera settings to capture images at 1,024 × 1,024 pixel size, at a scan speed of 8. Maintain these settings for the entire experiment. Optimization is required for each biological replicate.

e. Right after adding treatments, capture fluorescence images of the soma compartment of the microfluidics with excitation at 488 nm and 555 nm to capture CTB and PI, respectively, at 0 h.

f. Return the cells to the incubator for 24 h. Alternatively, for continuous live imaging, maintain the cells in the live imaging chamber on the microscope and capture images at different time points appropriate for the experiment.

g. Image the live cells in the soma compartment as mentioned above at the 24 h time point.

h. Once live imaging experiments are completed, fix cells with 4% paraformaldehyde solution while keeping the microfluidic device attached to the coverslip, if further immunolabeling post-fixation is intended. Then, the microfluidic device can be removed for further processing of the coverslips.

## Data analysis

This experimental protocol can be applied to understand the effects of axon-specific stimulation with ligands of choice on cell death, as PI does not incorporate into live cells. Therefore, we may assess the effect of compartmental treatments on neurons that are labeled with the retrograde tracer CTB but not PI at 0 h, by analyzing PI incorporation in these neurons at 24 h. CTB-positive neurons that do not incorporate PI in their nucleus at 24 h are considered surviving neurons, whereas CTB and PI double-positive neurons are considered dying neurons in response to the treatment (Figure 3a, b).

The retrograde degenerative effect of axonally sourced proneurotrophins in BFCNs was reported by Dasgupta et al. [8] using this model system and live imaging analysis. In this study, axonal treatment of BFCN microfluidic cultures with the proneurotrophins proNGF and proBDNF resulted in retrograde degeneration, in comparison to untreated control cells [8]. Live images of the entire soma compartment, CTB, and PI channels, were captured at 10× in an LSM 510 confocal microscope at 0 and 24 h. Microfluidic chambers with a minimum of 50 CTB-positive cells were considered for the analysis. Each treatment in every biological replicate had two technical replicates averaged for the final analysis. Quantification was done in ImageJ by counting PI in CTB+ve cells, which was normalized as a percentage of PI+CTB+ cells over CTB+ve cells [8]. A higher percentage indicates an increase in cell death.

## Validation of protocol

This protocol was used to establish the neurodegenerative effects of axon-specific stimulation of basal forebrain neurons with proneurotrophins by Dasgupta et al. [8]. In this study, it was shown that cortical injury promotes a retrograde degeneration of basal forebrain neurons through p75NTR. Moreover, it was established that cortical injury leads to an induction of proneurotrophins in the cortex but not in the basal forebrain, suggesting that p75NTR binds proneurotrophins at the BFCN axon terminals that innervate the cortex, which leads to retrograde degeneration. In vitro live imaging of microfluidic BFCN cultures using the described protocol showed that axon-specific proneurotrophin stimulation for 24 h promoted cell death [8], further supporting the hypothesis that axonally sourced proneurotrophins are sufficient to promote retrograde degeneration of BFCNs.

## General notes and troubleshooting

**A. Microfluidic chamber preparation**

1. Instead of making in-house devices, commercial silicone microfluidic devices may be purchased from Xona Microfluidics (XC450), which can be used with the same method.

2. The PDMS mixture should completely fill the mold during chamber preparation, as less PDMS will lead to the formation of a device with less thickness, making it too malleable and prone to breaking even though the negative relief of the microfluidic device will be functional.

3. The percentage of PDMS base to curing agent may be changed to produce a device with more or less elasticity. However, the proportion used was optimized in-house to have the most effective devices, which have adequate elasticity to form a complete seal with the glass coverslip, while not being too elastic, which hinders releasing the coverslips from the chambers for postprocessing such as immunostaining.

4. Post-rotating the PDMS mixture will have a lot of bubbles. Though the long desiccation process should help remove all the bubbles over time, spinning down the mixture in a centrifuge may aid in removing bubbles more effectively before the PDMS is poured into the molds, as well as shorten the duration of the desiccation step.

5. While creating media reservoirs in freshly prepared microfluidic devices, place punchers as close as possible to the edges of the compartments to ensure an open channel for media between the compartments. Using a sharp scalpel, the edge leading into the compartment can be shaved slightly to increase the opening, which reduces the fluid pressure and allows smooth media flow.

6. To maintain a water-tight seal between the microfluidic chamber and the coverslip it is placed on, both need to be dry at the time of assembly, as any moisture on either leads to a break in the seal, which hinders axon–soma separation.

**B. Basal forebrain dissection and live imaging**

1. It is important to have a minimum concentration of cells of 2–3 million/mL in the plate microfluidics. The total number of cells plated (50,000 cells) should be optimally present in a volume of 15 μL. A minimum of 8 μL to a maximum of 30 μL media volume is advisable. Below the minimum volume, cells may not optimally pass through the soma compartment and be concentrated on one side. Above the maximum volume, the cell density is too dilute to populate the soma compartment and may flow through to the other side. The aim is to condense the cell plating in the soma compartment and not the reservoirs.

2. In case of low cell concentration, collect cells in a sterile 15 mL Falcon tube and add 8 mL of media (SFM + 1% B27) to the cells. Centrifuge the cells at 400× *g* for 5 min at 4 °C to collect the cells in a pellet. Carefully remove the supernatant and resuspend the cells in an appropriate volume of media to obtain the optimal cell concentration as discussed above. Follow up with plating cells as described in the procedure (step A3c).

3. Methods of enzymatic dissociation such as trypsinization or DNase treatment may be chosen if the quality of the cell culture is being affected by the mechanical dissociation step. However, at this developmental age, the basal forebrain tissue is soft and should ideally not require it.

4. Incubation time after treating with CTB can be extended from 5 h to overnight in case the CTB signal is not prominent after 5 h to allow transport of the CTB cargo to the soma from the axon.
